# Comprehensive evaluation of diabetes subtypes in a European cohort reveals stronger differences of lifestyle, education and psychosocial parameters compared to metabolic or inflammatory factors

**DOI:** 10.1186/s12933-025-02660-5

**Published:** 2025-02-28

**Authors:** Nathalie Rohmann, Johannes Epe, Corinna Geisler, Kristina Schlicht, Kathrin Türk, Katharina Hartmann, Lucy Kruse, Julia Koppenhagen, Ahmad Yusuf Kohestani, Tanja Adam, Corinna Bang, Andre Franke, Dominik M. Schulte, Tim Hollstein, Matthias Laudes

**Affiliations:** 1 Institute of Diabetes and Clinical Metabolic Research, University Medical Center Schleswig-Holstein (UKSH) - Campus Kiel, Düsternbrooker Weg 17, 24105 Kiel, Germany; 2 Division of Endocrinology, Diabetes and Clinical Nutrition, Department of Internal Medicine I, University Medical Center Schleswig-Holstein - Campus Kiel, Kiel, Germany; 3 Department of Nutrition and Movement Sciences, School of Nutrition and Translational Research in Metabolism, Maastricht University, Maastricht, The Netherlands; 4 Institute of Clinical Molecular Biology, Kiel University and University Medical Center Schleswig-Holstein - Campus Kiel, Kiel, Germany

**Keywords:** Diabetes subtypes, Education, Diet, Exercise, Gut microbiota, Secondary disease risk

## Abstract

**Background:**

The traditional binary classification of diabetes into Type 1 and Type 2 fails to capture the heterogeneity among diabetes patients. This study aims to identify and characterize diabetes subtypes within the German FoCus cohort, using the ANDIS cohort's classification framework, and to explore subtype-specific variations in metabolic markers, gut microbiota, lifestyle, social factors, and comorbidities.

**Methods:**

We utilized data from 416 participants (208 with diabetes and 208 matched metabolically healthy controls) from the German FoCus cohort. Participants were classified into five subtypes: severe autoimmune diabetes (SAID)-like, severe insulin-deficient diabetes (SIDD)-like, severe insulin-resistant diabetes (SIRD)-like, mild obesity-related diabetes (MOD)-like, and mild age-related diabetes (MARD)-like. Comprehensive characterization included anthropometric measurements, dietary and physical activity questionnaires, blood biomarker analysis, and gut microbiota profiling.

**Results:**

The subtype distribution in the FoCus cohort accounted to SAID-like: 2.84%, SIDD-like: 30.81%, SIRD-like: 32.23%, MOD-like: 17.54%, MARD-like: 16.59%. Of interest, inflammatory markers (C-reactive protein (CRP) and Interleukin-6 (IL-6)) and glucagon-like peptide-1 (GLP-1) levels were similarly elevated across all subtypes compared to controls, indicating common aspects in Type 2 diabetes molecular pathology despite different clinical phenotypes. While the gut microbiota and dietary patterns only showed minor differences, smoking status, sleep duration, physical activity and psychological aspects varied significantly between the subtypes. In addition, we observed a lower educational status especially for SIDD-like and SIRD-like groups, which should be considered in establishing future diabetes-related patient education programs. In respect to the development of cardio-metabolic comorbidities, we observe not only significant differences in the presence of the diseases but also for their age-of onset, highlighting the need for early preventive intervention strategies.

**Conclusions:**

The study validates the ANDIS classification framework's applicability not only at the time point of manifestation but also in cohorts with pre-existing diabetes. While we did not find major differences regarding the classical metabolic, microbial and nutritional parameters, we identified several significant associations with lifestyle factors. Our findings underscore the importance of personalized, subtype-specific therapies not solely focusing on anthropometric and laboratory markers but comprehensively addressing the patient’s own personality and situation of life.

**Supplementary Information:**

The online version contains supplementary material available at 10.1186/s12933-025-02660-5.

## Background

Traditionally, diabetes has been classified into two main types: Type 1, characterized by absolute insulin deficiency due to an autoimmune reaction, and Type 2, marked by a progressive loss of sufficient insulin production alongside the development of insulin resistance. However, recent research suggests that this binary classification inadequately captures the heterogeneity of diabetes phenotypes [1–3].

The increasing prevalence of diabetes-related complications, which contribute to approximately 21% of deaths in Germany [4], underscores the urgent need for earlier detection and targeted treatment. Efforts to develop a more nuanced classification system for Type 2 diabetes have led to the identification of five subtypes (also referred to as endotypes or subclasses) within the Swedish ANDIS cohort: severe autoimmune diabetes (SAID), severe insulin-deficient diabetes (SIDD), severe insulin-resistant diabetes (SIRD), mild obesity-related diabetes (MOD), and mild age-related diabetes (MARD) [5]. These subtypes are defined based on clinical measurements, such as GAD-antibody for autoimmunity, HbA1c for glycemic control, Homeostatic Model Assessment (HOMA) for insulin resistance (-IR) and beta-cell function (-b), age-at-disease-onset and obesity presence and show associations with specific complications [6]. Studies on this new subtyping approach aim to enhance the understanding necessary for effective therapy and prevention strategies [5, 6].

In our study, we want to identify these diabetes subtypes within the existing FoCus cohort, using the ANDIS cohort as a reference framework but adapting the parameters to suit our dataset. Unlike the ANDIS cohort, which focuses on the classification of newly diagnosed diabetes cases, our approach utilizes a pre-existing cohort. This methodological adaptation allows us to explore the classification’s applicability and dynamic in a different cohort context.

Following the identification of these ANDIS-like subtypes, we aim to conduct a comprehensive characterization covering metabolomic markers, microbial composition, nutrition, and lifestyle factors. Our study assesses extensive health-related data from the Kiel FoCus cohort, comprising individuals with diabetes (n = 209) and metabolically healthy control subjects (n = 209). This dataset includes information on health status, daily medication intake, dietary habits, physical activity, sleep patterns, various serum biomarkers, and fecal microbiota diversity. By integrating insights from these diverse research domains, this study aims to develop hypotheses that contribute to the optimization of diabetes therapy, specifically targeting a personalized, subtype-specific approach. Consequently, our objective is to attain a more nuanced understanding of diabetes subtypes in a pre-existing cohort, focusing on previously underexplored facets, and thereby contributing to a more comprehensive profile for each subtype.

## Methods

### Study design and population

For the identification and characterization of ANDIS-like diabetes subtypes we used data from the German population-based “Food Chain Plus” (FoCus) cohort, previously described by Geisler et al. [7]. Subjects who reported a previous diabetes diagnosis were classified into the five subtypes based on BMI, age-at-diabetes-onset in years, Homeostatic Model Assessment of insulin resistance (HOMA-IR) and -of beta-cell function (HOMA-beta) values, diabetes duration in years, diabetes therapy (none/diet, oral antidiabetics (OAD) or insulin) and previous self-reported Type 1 or 2 diabetes. Individual control groups were formed for each subtype consisting of metabolically healthy controls matched to the closest age- and sex-match. Participants were defined as metabolically healthy based on (1) Body Mass Index (BMI) within normal range (18.5–25 kg/m^2^); (2) glucose, insulin, triglyceride, total cholesterol, C-reactive protein (CRP) and Interleukin 6 (IL-6) levels within normal ranges and (3) absence of cardio-metabolic disorders (diabetes mellitus, chronic heart failure, coronary artery disease, myocardial infarction or atrial fibrillation).

Characterization of diabetes subtypes was subsequently performed including psychosocial parameters, dietary and physical activity questionnaires, sleep duration, smoking habits as external factors and blood biomarkers, gut microbiota and secondary disease risk assessment as measures of the metabolic phenotype.

**Fig. 1:**
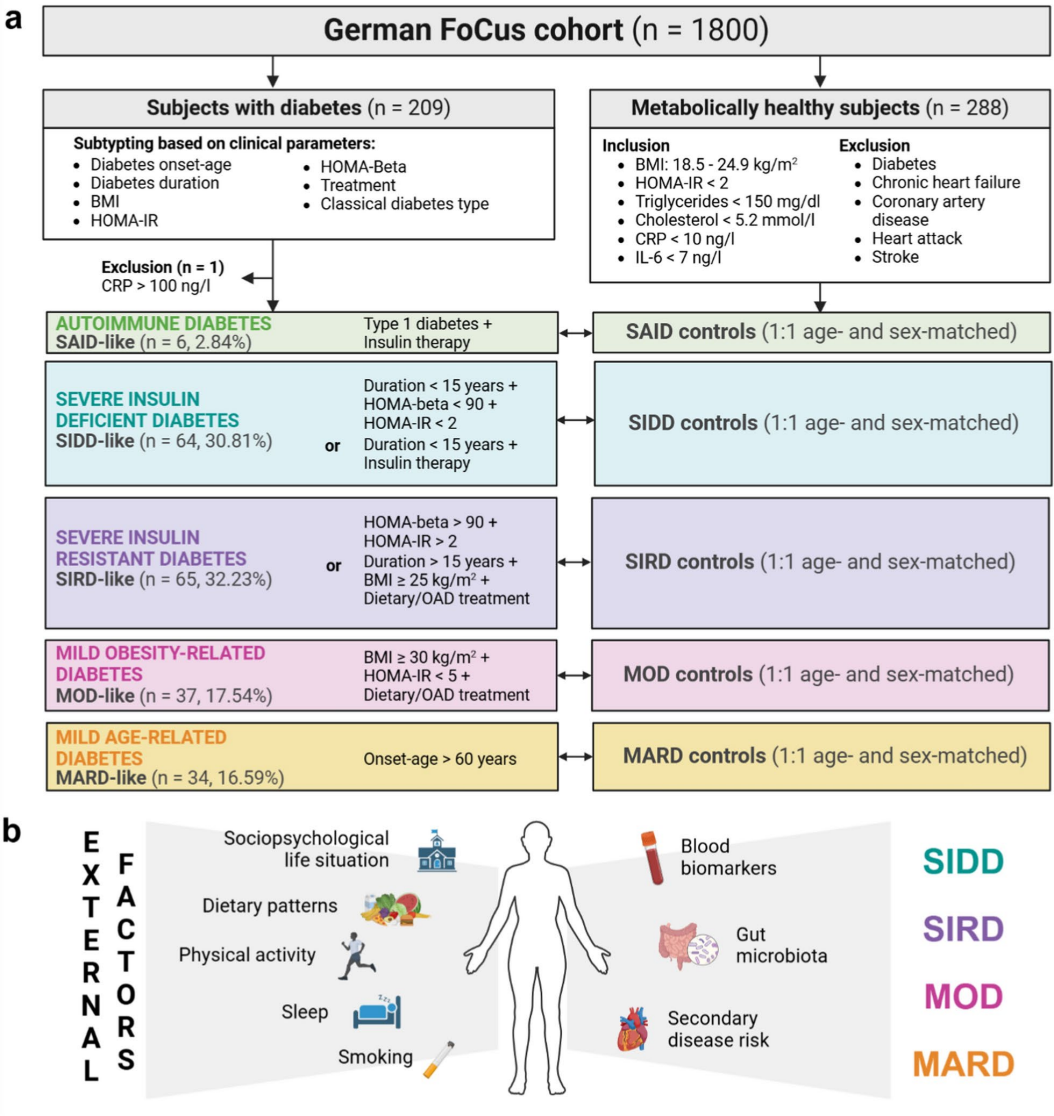
Study population and overview of diabetes subtype characterization. **a** Subjects from the German FoCus cohort who reported a previous diabetes diagnosis (n = 209) were classified into five subtypes of the following characteristics: severe autoimmune diabetes (SAID), severe insulin-deficient diabetes (SIDD), severe insulin-resistant diabetes (SIRD), mild obesity-related diabetes (MOD), mild age-related diabetes (MARD) based on BMI, age-at-diabetes-onset in years, Homeostatic Model Assessment of insulin resistance (HOMA-IR) and -of beta-cell function (HOMA-beta) values, diabetes duration in years, diabetes therapy (none/diet, oral antidiabetics (OAD) or insulin) and previous self-reported Type 1 or Type 2 diabetes diagnosis. Subjects with diabetes were each paired with metabolically healthy controls using the closest age- and sex-match. Each subtype was paired with an individual control group. Participants, whose Body Mass Index (BMI) was within normal range (18.5–25 kg/m^2^), whose glucose, insulin, triglyceride, total cholesterol, C-reactive protein (CRP) and Interleukin 6 (IL-6) levels were within normal ranges and who did not report about a cardio-metabolic disorder (diabetes mellitus, chronic heart failure, coronary artery disease, myocardial infarction or atrial fibrillation), were defined as metabolically healthy. **b** A comprehensive characterization of diabetes subtypes was subsequently performed including psychosocial parameters, dietary and physical activity questionnaires, sleep duration, smoking habits as external factors and blood biomarker analysis, gut microbiota profiling and secondary disease risk assessment as measures of the metabolic phenotype

Details on study design and population are provided in Fig. 1.

### Data collection

Data collection for the FoCus cohort comprised a physical examination, a comprehensive medical questionnaire, analysis of blood and stool samples, and another semi-quantitative food frequency questionnaire.

#### Anthropometry

Height, weight, waist and hip circumference were collected through physical examination conducted by medical professionals at the University Medical Center Schleswig–Holstein (UKSH). From these measurements, Body Mass Index (BMI) and waist-hip ratio (WHR) were calculated. Maximum grip strength was determined from triplicate measurements of each hand using a hand dynamometer.

#### Health status and medical history

A medical questionnaire provides information on the current health status through structured question blocks, including the presence of chronic diseases, medications, and, where applicable, additional relevant specifications (e.g. age of onset, type and treatment). Additionally, various lifestyle factors such as smoking behavior were asked through the questionnaire.

#### Biochemistry

Blood samples from each participant were collected at the study site by medical staff. Clinical metabolic markers were quantified at the central laboratory of the UKSH, Kiel, Germany. Additional biomarkers were measured at the Institute of Diabetes and Clinical Metabolic Research using commercial ELISAs: Glucagon-like peptide 1 (GLP-1, Mercodia, 10-1278-01), soluble Dipeptidyl Peptidase-4 (sDPP-4, Abcam, ab222872), WNT Family Member 5A (WNT5a, MyBioScource, MBS162566) and secreted frizzled related protein 5 (SFRP5, Cloud-Clone Corp, SEC842Hu). Homeostatic Model Assessment for insulin resistance and beta-cell function were calculated from glucose and insulin values: HOMA-IR = glucose × insulin/ 405, HOMA-beta = 360* insulin/(glucose-63).

#### Gut microbiota

Fecal samples from study participants were collected at home using standard stool collection tubes and sent to the study center where they were stored at -80 °C until further use. Sample processing, including DNA extraction, and 16S rRNA sequencing of the V1-V2 region were performed by the Institute of Clinical Molecular Biology at Kiel University. Procedures were previously described in detail by [8, 9].

#### Diet and physical activity

A questionnaire regarding dietary behavior over the past 12 months [10–12] has been used for the evaluation of dietary intake. For the evaluation of diet quality, we calculated the Healthy Eating Index (HEI-EPIC) using the questionnaire data [13]. The calculation process was previously described in detail [14]. The questionnaire also includes information on physical activity and sleep.

### Statistical analysis

Statistical analysis was performed using R (Core Team, 2022, version 4.1.2) and RStudio (version 2023.09.1). For case–control matching, the “MatchIt” package [15] was applied; for data visualization, the packages “ggplot2” and “ggpubr” [16] were used.

#### Analysis of blood biomarkers, diet, lifestyle and -situation parameters

The median [25.-75. percentile] are used to display continuous variables. Pairwise univariate comparisons between subtypes and between each subtype and their respective control group were performed using Wilcoxon-tests. Categorical variables are displayed as prevalence (95% confidence interval). Significant differences between categorical variables were examined using Chi^2^-tests.

#### Analysis of the gut microbiota

Gut microbiota analysis covered the calculation of alpha diversity Shannon and Chao1 indices at ASV level using the “vegan” package in R [17]. A core measurable microbiota (CMM) was extracted including all ASVs with a prevalence ≥ 40% and relative abundance ≥ 0.5%. The CMM was used for the evaluation of relative microbial composition at genus level. Statistical significance between subtypes and each subtype and the control group was tested using pairwise Wilcoxon-tests. Beta diversity was evaluated with the Bray–Curtis dissimilarity and pairwise Permutational multivariate analysis of variances (PERMANOVA) between subtypes and between subtypeand control group. This was executed with the “vegan” and “pairwiseAdonis” [18] packages in R.

#### Analysis of secondary disease probability

For the evaluation of secondary disease probability in relation to diabetes subtypes, a Cox proportional hazards regression analysis (Cox-PH-regression) was individually applied for each evaluated secondary cardio-metabolic disease. Age-at-disease-onset served as dependent variable, diabetes subtypes as independent variable. For participants with diabetes but without the secondary disease, the age-at-examination was used as censoring parameter. Kaplan–Meier curves were constructed to estimate time until secondary disease onset. The R “survival” package [19] and the “coxph” function were utilized for this analysis.

A significance level of alpha < 0.05 was applied for all tests.

## Results

### Identification of ANDIS-like diabetes subtypes in the FoCus cohort

With our study, we aim to extend the diabetes subtyping established by Ahlqvist and colleagues [5] to assess diabetes subtypes in a cross-sectional cohort design. We used participants with previous diabetes diagnosis from the FoCus cohort. These were, with some adaptation, assigned to the five subclasses suggested by Ahlquvist et al*.* [5] (see Fig. 1 for details on categorization).

As displayed in Table 1, profiles of the identified subtypes for disease severity, insulin resistance and -deficiency show similar signatures as the subtypes described in the ANDIS cohort, despite different classification criteria and varying disease durations. Still, we are aware that by altering the inclusion criteria, we cannot exactly reproduce the ANDIS-subtypes. To ensure clear differentiation from the original subtypes, we added the suffix “-like'' to subtype designation.

**Table 1:** Characterization of five ANDIS-like diabetes subtypes in the Kiel FoCus cohort

	SAID-like subtype	SIDD-like subtype	SIRD-like subtype	MOD-like subtype	MARD-like subtype	*p*-value^a^
Subjects, n (%)	6 (2.84)	64 (30.81)	66 (32.23)	37 (17.54)	35 (16.59)	–
Female sex, n (%)	3 (50)	32 (50)	36 (54.55)	27 (72.97)	20 (57.14)	0.27
Age at examination, years	46.50 [43.75–48.50]	53.5 [47; 59.25]	55 [44.5; 63.75]	60 [49; 64]	70 [65.5; 73]	< 10^–5^
Age at diagnosis, years	25 [18; 32.5]	47 [39; 52]	45 [40; 50]	51 [43.5; 59]	64 [63; 67]	< 10^–5^
Disease duration, years	20 [11; 35.5]	8 [4.75; 12]	6 [4; 18]	5 [1.5; 7]	2 [1; 7.5]	1.41 × 10^–5^
BMI, kg/m^2^	33.88 [25.3–45.81]	42.17 [33.04; 50.38]	42.11 [32.50; 48.61]	38.91 [32.93; 42.68]	30.04 [26.32; 36.64]	< 10^–5^
Waist measure, cm	90 [84.75–101.25]	118 [110; 131]	122 [110; 137]	118 [110.5; 123]	107.5 [96; 121.5]	2.13 × 10^–3^
Waist-hip-ratio	0.84 [0.8–0.89]	0.96 [0.92; 1.01]	0.98 [0.93; 1.02]	0.94 [0.91; 1]	0.95 [0.90; 1]	1.52 × 10^–2^
HOMA-beta	0.81 [0.59–1.1]	95.05 [64.56; 192.16]	174.46 [101.45; 341.18]	125.22 [75.53; 202.66]	111.39 [54.75; 188.22]	3.41 × 10^–5^
HOMA-IR	0.21 [0.09–0.54]	12.26 [5.92; 24.35]	10.92 [5.85; 16.87]	3.71 [2.50; 4.36]	5.20 [3.52; 7.92]	< 10^–5^
Glucose, mg/dl	189 [174–282]	151.5 [124; 207.5]	121 [109; 162]	102 [93.75; 115.75]	118 [106; 138]	< 10^–5^
Insulin, µU/l	0.3 [0.2; 0.7]	30 [14.9; 54]	34.9 [20.8; 50.9]	14.75 [9.43; 17.28]	17.70 [9.90; 27.25]	< 10^–5^
Diabetes treatment, % (95% CI)						< 10^–5^
None	0	3.12 (0.86–10.7)	6.06 (2.38–14.57)	13.51 (5.91–27.98)	18.18 (8.61–34.39)	
Diet	0	12.5 (6.47–22.77)	10.61 (5.23–20.31)	13.51 (5.91–27.98)	15.15 (6.65–30.92)	
OAD	16.67 (0.88–63.65)	25 (16.01–36.82)	56.06 (44.08–67.37)	72.97 (57.02–84.6)	63.64 (46.62–77.81)	
Insulin	100 (60.97–100)	31.25 (21.23–43.39)	13.64 (7.34–23.93)	0	6.06 (1.68–19.61)	

Continuous data is displayed as median [Interquartile Range]; categorical data as percentage (95% confidence intervals)

^a^Statistical significance between diabetes subtypes was tested using Chi^2^-tests for categorical variables and Kruskal–Wallis-tests for continuous variables

BMI, Body Mass Index; HOMA-IR, Homeostatic Model Assessment of Insulin Resistance; HOMA-beta, Homeostatic Model Assessment of beta-cell function

Each subject with diabetes was assigned a metabolically healthy control resulting in five subclass-specific control groups and a total study population of 418 participants. Characterization of these control groups along comprehensive comparison of each subtype and their control group are provided as Supplement.

One participant in the SIDD-like subtype displayed a CRP > 100 ng/l and was therefore excluded from further analyses. Since there were only six participants in the SAID-like subtype, this subtype has not been further evaluated going forward.

### Evaluation of blood biomarkers in relation to the FoCus diabetes subtypes

Following the successful establishment of diabetes subtypes within our cohort, we intend to further characterize these subtypes. In this subsequent phase, we aim to identify biomarkers strengthening the identified subtype-specific differences.

#### Lipid metabolism markers

**Fig. 2:**
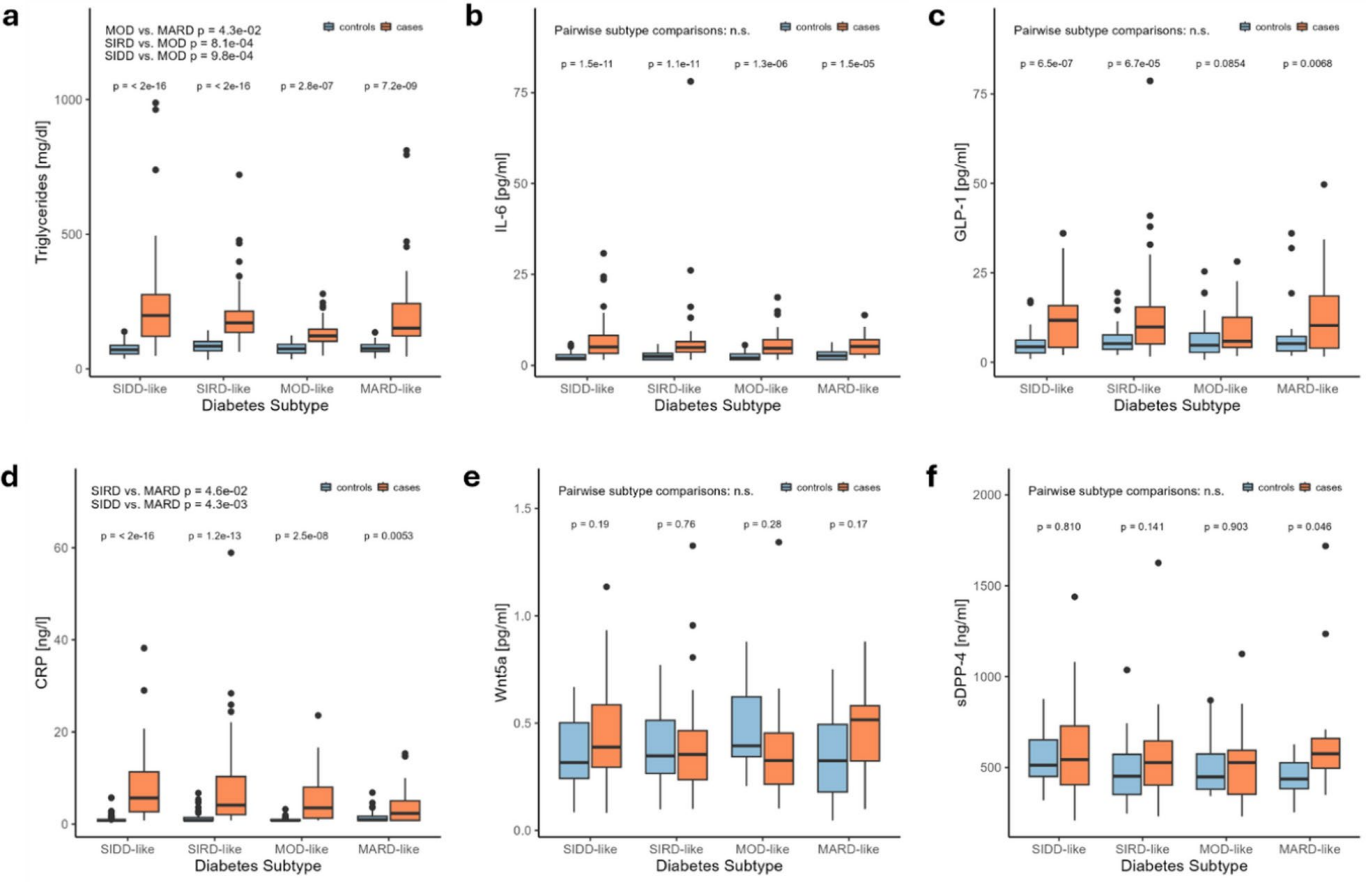
Comparison of blood biomarkers between diabetes subtypes and between subtype cases and metabolically healthy control subjects. Statistical significance between different diabetes subtypes and between diabetes subtypes and their respective control groups was determined using pairwise Wilcoxon tests with a significance level of *p* < 0.05. Number of observations: Triglycerides/CRP/IL-6—400, GLP-1—370, Wnt5a—180, sDPP-4—184. Abbreviations: CRP = C-reactive protein, IL-6 = interleukin 6, GLP-1 = Glucagon-like peptide 1, sDPP-4 = soluble Dipeptidylpeptidase-4, SAID = Severe Autoimmune Diabetes, SIDD = Severe Insulin Deficient Diabetes, SIRD = Severe Insulin Resistant Diabetes, MOD = Moderate Obesity-related Diabetes, MARD = Moderate Age-Related Diabetes

Strong differences in triglyceride levels are notable between each subtype and their respective control group. The MOD-like subtype displays significantly higher levels compared to the corresponding healthy controls, but lower levels compared to the SIDD- and SAID subjects. (Fig. 2a). This effect is also present in people who do not take lipid-lowering medication. In turn, total cholesterol levels do not differ between subtypes and are not altered by diabetes compared to metabolically healthy controls (data not shown).

#### Inflammation markers

Increased levels of CRP and IL-6 are present in all diabetes subtypes compared to their control groups (Fig. 2b + d). Among diabetes cases, the MARD-like subtype displays the lowest CRP values reaching statistical significance in comparison to the SIRD-like and the SIDD-like subtype while no differences can be seen between IL-6 values. WNT5a (Fig. 2e) and SFRP5 (data not shown) are available for investigation of adipose tissue-driven metabolic inflammation but do not display any sort of impairment.

#### Incretin system markers

Increased serum GLP-1 is present in all subtypes compared to control groups, however, not reaching statistical significance for the MOD-like subtype. No differences were found among the four subtypes (Fig. 2c). While no differences between sDPP-4 between cases and controls can be detected for the SIDD-like, SIRD-like and MOD-like subtypes, there is a slight increase in sDPP-4 in the MARD-like subtype (Fig. 2f). To exclude potential confounding from medication use, the effects of administered GLP-1 analogs and DPP-4 inhibitors on serum GLP-1 and DPP-4 levels were analyzed confirming that neither serum levels are significantly altered by these medications.

### Evaluation of lifestyle determinants in relation to the FoCus diabetes subtypes

To explore the participants' lifestyle, an examination of smoking habits, physical activity and fitness, as well as sleep duration was performed. Data was available for N = 405 (smoking habit), N = 399 (physical fitness) and N = 385 (physical activity and sleep).

#### Smoking habits

**Fig. 3:**
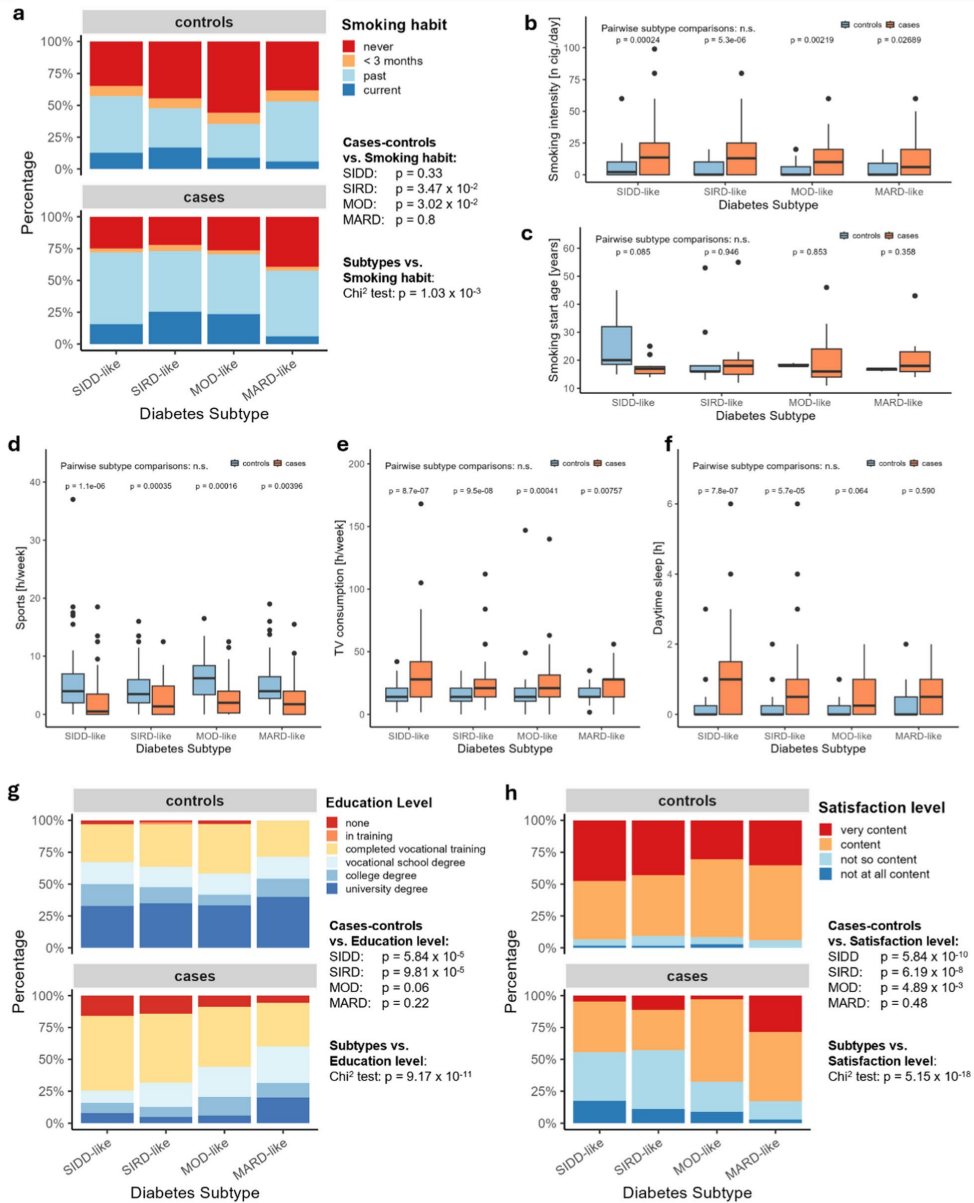
Comparison of lifestyle, highest education level and life satisfaction between the FoCus diabetes subtypes and between subtype cases and metabolically healthy control subjects. Significance between different diabetes subtypes and between diabetes subtypes and their respective control groups was determined using Chi^2^-tests for categorical variables and pairwise Wilcoxon tests for continuous variables with a significance level of *p* < 0.05. Number of observations: Smoking habit—390, smoking intensity—290, smoking start age—68, education level—393, satisfaction level—392. Abbreviations: SIDD = Severe Insulin Deficient Diabetes, SIRD = Severe Insulin Resistant Diabetes, MOD = Moderate Obesity Diabetes, MARD = Moderate Age-Related Diabetes, n cig. = number of cigarettes

The analysis of smoking habits (never smoked vs. smoked in the past vs. smoked for less than 3 months vs. currently smoking) displays significant differences between subtypes with the MARD-like subtype having the highest proportion of participants who never smoked and the lowest proportion of participants who are currently smoking. In return, the SIRD- and MOD-like subtypes display similarly high rates of current smokers, with significant differences in comparison to controls (Fig. 3a). Furthermore, participants with diabetes reported significantly higher daily cigarette numbers than healthy controls, independent of subtype (Fig. 3b). No differences can be seen regarding the age of beginning to smoke (Fig. 3c).

#### Physical activity and -fitness

Separate analyses were performed for everyday- and sports activities. No differences can be observed in everyday activities between cases and controls, while there is some variation among subtypes with the SIRD-like subtype stating the lowest rates of everyday activities (SIRD vs. MARD: *p* = 3.8 × 10^–2^). Subtype variation is not apparent regarding sports activities. Yet, individuals with diabetes engage in significantly less sports than healthy controls overall (Fig. 3d). The examination of television watching reveals no significant differences between subtypes. However, significant differences can be seen between all subtypes and their control groups (Fig. 3e). Analysis of maximum grip strength as marker for physical fitness does not indicate any differences, neither among subtypes, nor in comparison with healthy control subjects (data not shown).

Sleep: Notable differences emerge regarding daytime sleep duration with significantly longer duration in the severe SIDD-like and SIRD-like subtypes compared to controls (Fig. 3f). This also translates to longer overall sleep duration per 24-h cycle (SIDD-like vs. controls: *p* = 1.1 × 10^–2^; SIRD-like vs. controls: *p* = 2.7 × 10^–2^). No differences are present regarding nighttime sleep duration (data not shown).

### Evaluation of social factors in relation to the FoCus subtypes

In the next step, we want to assess how the diabetes subtypes correlate with the individuals’ life situation measured by educational status and objective satisfaction with life.

#### Education

A clear distinction among the educational status is evident comparing the SIDD-like and SIRD-like subtypes to control groups (Fig. 3g). Controls display higher proportions of college graduates, while the SIDD-like and SIRD-like subtypes are characterized by the highest proportions of participants without any educational training. Comparison of education between subtypes also displays significant differences with the MARD-like subtype showing the smallest proportion of participants without educational training and the highest proportion of college graduates (Fig. 3g).

#### Life satisfaction

As displayed in Fig. 3h, the examination of life satisfaction also yields significant results. In the control groups, most participants reported to be content or very content in life, while over 50% of participants in the SIDD-like and SIRD-like report to be not (at all) content with their lives. Among subtypes, the MARD-like subtype displays the most content individuals. The MOD-like subtype reflects an in-between satisfaction status with slightly lower levels than the MARD- but higher than the SIDD- and SIRD-like subtypes.

### Evaluation of the gut microbiota and diet in relation to the FoCus diabetes subtypes

The following analysis focuses on examining the diversity and composition of the gut microbiota as well as dietary intake in association to diabetes subtypes. Gut microbiota sequencing was available for n = 351 subjects, dietary questionnaires for n = 385.

#### Gut microbiota

**Fig. 4:**
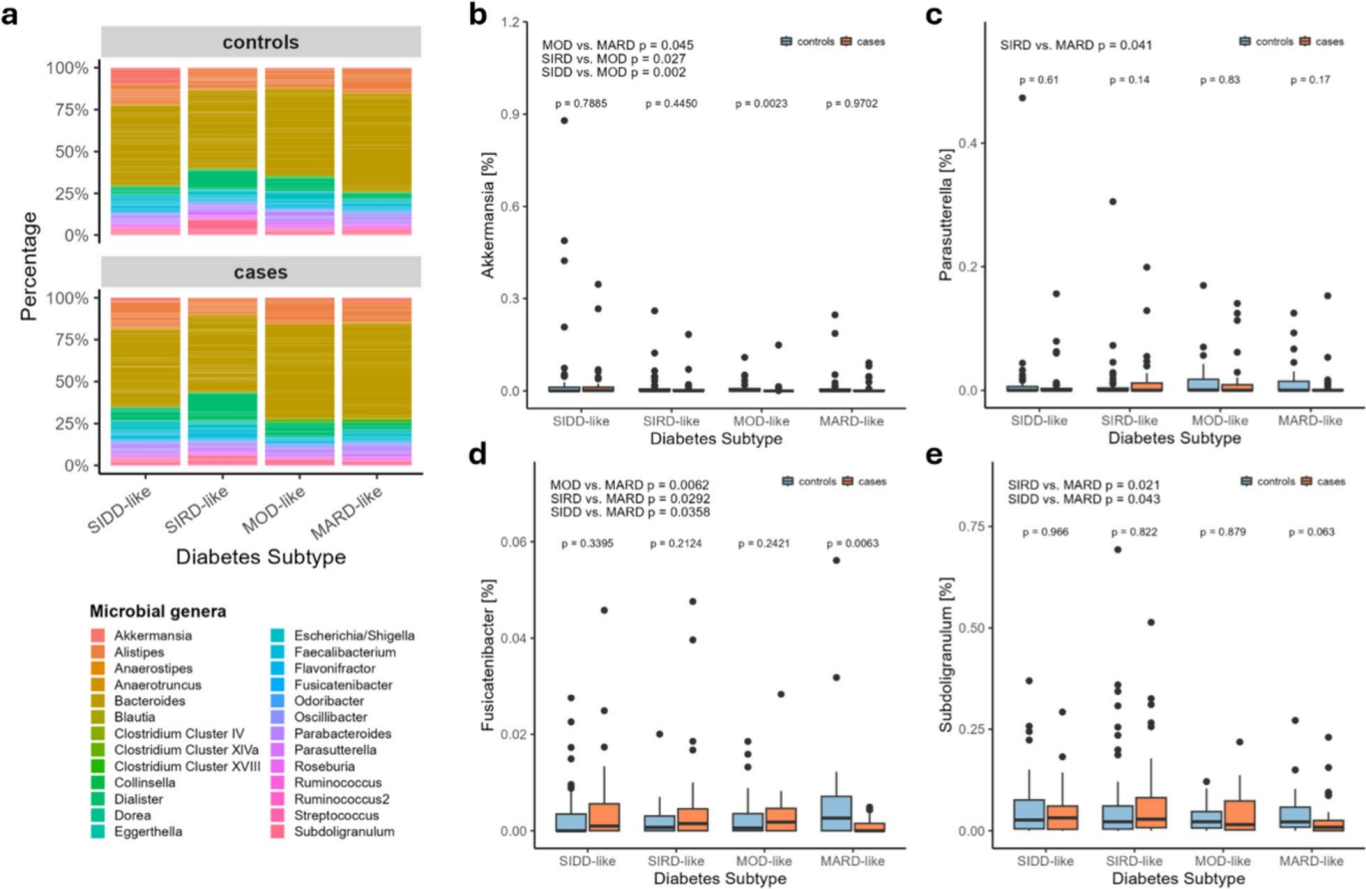
Comparison of core gut microbiota composition between diabetes subtypes and between subtype cases and metabolically healthy control subjects. Statistical significance between different diabetes subtypes and between diabetes subtypes and their respective control groups was determined using pairwise Wilcoxon tests with a significance level of *p* < 0.05. Number of observations: 319. Abbreviations: SIDD = Severe Insulin Deficient Diabetes, SIRD = Severe Insulin Resistant Diabetes, MOD = Moderate Obesity-related Diabetes, MARD = Moderate Age-Related Diabetes

Alpha diversity Shannon and Chao1 indices do not exhibit alterations—neither between diabetes cases and controls nor for a specific subtype. Likewise, the Bray–Curtis dissimilarity approach and pairwise PERMANOVA tests do not indicate significant differences in beta diversity of any sort (diversity data not shown). Microbial composition is evaluated using the relative abundance of the core microbiota (CMM, 90 ASVs of 26 genera). Overall composition of the CMM and comparisons of the relative abundance that show significant differences are displayed in Fig. 4. This comprises the genera *Akkermansia* with decreased levels in the MOD-like subtype (Fig. 4b), *Parasutterella* with variation among the severe subtypes (Fig. 4c) as well as *Fusicatenibacter* and *Subdoligranulum* both decreased in the MARD-like subtype (Fig. 4c + d).

#### Diet

**Fig. 5:**
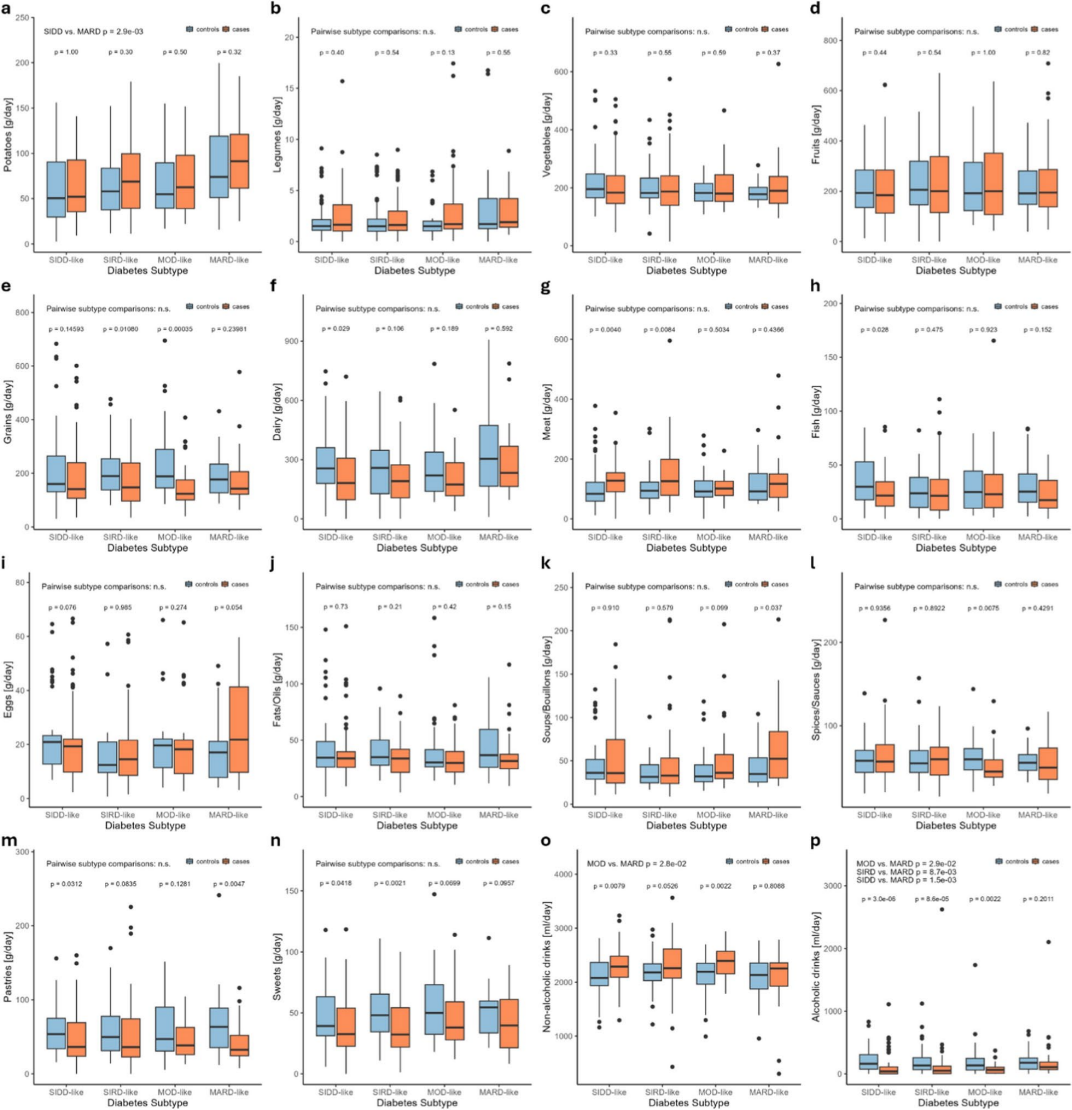
Comparison of diet composition between diabetes subtypes and between subtype cases and metabolically healthy control subjects. The MARD-like subtype reported a higher intake of potatoes in comparison to the SIDD-like subtype (**a**) and of alcoholic drinks in comparison to all other subtypes (**p**). Regarding the case–control analysis, the SIDD-like subtype shows a higher intake of meat (**g**) and non-alcoholic drinks (**o**) and lower intake of dairy (**f**), fish (**h**), pastries (**m**) and alcoholic drinks (**p**); the SIRD-like subtype shows a higher intake of meat (**g**) and lower intake of grains (e) and alcoholic drinks (p); the MOD-like subtype shows a higher intake of non-alcoholic drinks (**o**) and lower intake of grains (**e**), spices/sauces (**l**) and alcoholic drinks (**p**); the MARD-like subtype shows a higher intake of soups/bouillons (**k**) and lower intake of pastries (**m**). Statistical significance between different diabetes subtypes and between diabetes subtypes and their respective control groups was determined using pairwise Wilcoxon tests with a significance level of *p* < 0.05. Number of observations: 385. Abbreviations: SIDD = Severe Insulin Deficient Diabetes, SIRD = Severe Insulin Resistant Diabetes, MOD = Moderate Obesity-related Diabetes, MARD = Moderate Age-Related Diabetes

The diet quality was evaluated using the Healthy Eating Index and does not differ significantly in-between subtypes or in comparison to healthy controls (data not shown). The comprehensive comparison of diet composition, which is provided in Fig. 5 also does not indicate a unique dietary pattern for each subtype but rather variations among singular food groups.

### Cardio-metabolic secondary diseases in association to diabetes subtypes

In this final analysis, we assess the risk for common cardio-metabolic diseases in association to diabetes subtypes. This analysis only includes subjects with diabetes and separately considers arterial hypertension, elevated blood lipids, cardio-vascular events (myocardial infarction, stroke, chronic heart failure and/or coronary artery disease) and gall stone development. Initially, we assessed the prevalence of these diseases revealing no significant differences between subtypes (see Table 2).

**Table 2:** Prevalence of secondary cardio-metabolic diseases in association to diabetes subtypes

	SIDD-like	SIRD-like	MOD-like	MARD-like	*p*-value
Arterial hypertension, % (95% CI)	90.63 (81.02–95.63)	80.0 (68.73–87.92)	83.33 (68.11–92.13)	85.71 (70.62–93.74)	0.47
Elevated blood lipids, % (95% CI)	54.69 (42.57–66.27)	49.23 (37.46–61.08)	52.78 (37.01–69.01)	51.43 (35.56–67.01)	0.9
Cardio-vascular events, % (95% CI)	20.31 (12.27–31.71)	27.69 (18.29–39.58)	22.22 (11.72–38.09)	34.29 (20.83–50.85)	0.4
Gall stones, % (95% CI)	25.0 (16.01–36.82)	24.62 (15.76–36.31)	27.78 (15.85–43.99)	14.29 (6.26–29.38)	0.61

Disease prevalence is displayed as percentage (95% confidence intervals). Statistical significance between diabetes subtypes was tested using Chi^2^-tests.

SIDD = Severe Insulin Deficient Diabetes, SIRD = Severe Insulin Resistant Diabetes, MOD = Moderate Obesity Diabetes, MARD = Moderate Age-Related Diabetes, CI = confidence interval

**Fig. 6:**
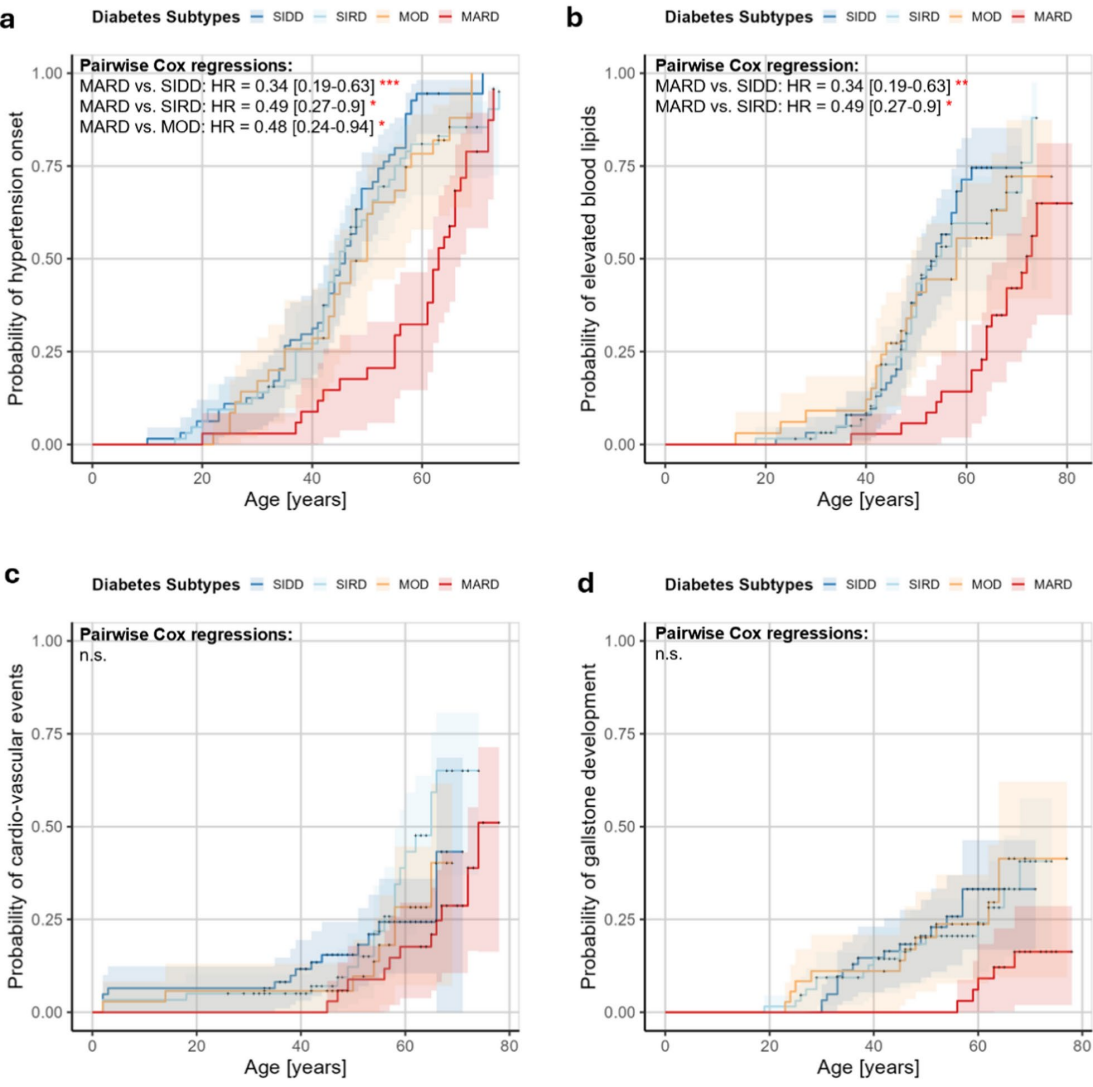
Cox-regression analysis of secondary disease development in relation to diabetes subtypes. Cox-regression models of secondary disease presence/absence and corresponding onset-age/age-at-examination were used to identify differences between diabetes subtypes with a significance level of *p* < 0.05. Exact p-values for **a** hypertension analyses: MARD versus SIDD: *p* = 5.17 × 10^–5^, MARD vs. SIRD: *p* = 1.36 × 10^–2^, MARD vs. MOD: *p* = 2.61 × 10^–2^ and **b** elevated blood lipids: MARD vs. SIDD: *p* = 1.35 × 10^–3^, MARD vs. SIRD: *p* = 1.09 × 10^–2^. Number of data points: hypertension– 188 (presence: 163; absence: 25), elevated blood lipids– 186 (presence: 102; absence: 84), cardio-vascular events– 191 (presence: 51, absence: 140), gall stone development– 187 (presence: 45; absence: 142). Abbreviations: SIDD = Severe Insulin Deficient Diabetes, SIRD = Severe Insulin Resistant Diabetes, MOD = Moderate Obesity-related Diabetes, MARD = Moderate Age-related Diabetes, HR = Hazard Ratio

As a next step, we combine information on the presence and onset-age of the secondary diseases in pairwise Cox-regression models. In case of disease absence, the age-at-examination was used, thereby censoring these control subjects. With this analysis, the probability of disease onset in association to diabetes subtypes and age can be estimated. Figure 6 displays the corresponding Kaplan–Meier curves with hazard ratios [95% confidence interval] and significance levels of comparisons with a *p*-value < 0.05.

In our study cohort, the MARD-like subtype is associated with later hypertension development in comparison to all other subtypes (Fig. 6a) and delayed development of elevated blood lipids in comparison to the severe SIDD-like and SIRD-like subtypes (Fig. 6b). No differences in secondary disease risk can be observed for cardio-vascular events or gall stones (Fig. 6c + d).

## Discussion

The classification of subtypes has become a central objective of diabetes research. Various approaches have been developed to refine this classification, with the attempt from the Swedish ANDIS cohort being most promising [5]. Building upon this, our study aimed to establish a similar subtype classification for the cross-sectional German FoCus cohort. Unlike the ANDIS cohort's focus on newly diagnosed cases, our study utilized a pre-existing cohort allowing us to assess the classification's applicability in a different cohort setting and utilize available data for further subtype characterization.

Our results confirmed the successful establishment of diabetes subtypes within the FoCus cohort, reflecting similar patterns of disease severity, insulin sensitivity, -resistance and age as observed in the ANDIS cohort [5, 6]. This validates the applicability of this classification framework to a pre-existing cohort. Notably, the distribution of subtypes in our cohort (SAID-like: 6 (2.84%), SIDD-like: 64 (30.81%), SIRD-like: 66 (32.23%), MOD-like: 37 (17.54%), MARD-like: 35 (16.59%)) differs from previous reports. This is likely due to the cohort setup, which partly targeted obese individuals.

We observed significant variations in lipid metabolism, inflammatory, and incretin system markers among the subtypes. Systemic inflammation markers CRP, IL-6, and the incretin hormone GLP-1 were elevated across all subtypes compared to controls. Low-grade chronic inflammation has been identified as risk factor for diabetes manifestation [20] and the similarity among subtypes suggests a common inflammatory basis. GLP-1 levels were also consistently high in diabetes with only slightly lower levels in the MOD-like subtype. This finding aligns with observations by Amato et al., who reported significant differences in GLP-1 levels among clusters of newly diagnosed diabetes, despite no differences in age, disease duration, anthropometric parameters, or insulin sensitivity [21]. Based on these observations, CRP, IL-6 and GLP-1 seem to be less suitable for differentiating between subtypes. Regarding lipid metabolism, the MOD-like subtype exhibited higher triglyceride levels compared to the corresponding healthy controls and lower levels compared to the SIRD-like subtypes, the latter might be related to the more severe metabolic rearrangement found in severe insulin resistant patients. Of interest, in our study the SIDD-like group also exhibited higher triglyceride levels compared to the MOD-like group, which is in contrast to the German Diabetes Study of Zaharia et al. (2019). This difference might be related to the longer disease duration as well as the higher body weight of the SIDD-like subjects in our cohort.

Subtypes displayed variation among food groups relevant in the context of diabetes, while overall diet quality was similar between individuals with diabetes and healthy controls proposing a general adherence to diabetes-related dietary recommendations and training [22]. Weber and colleagues also recently evaluated dietary patterns among diabetes subtypes and concluded no major differences in dietary behavior, but rather a relevant interplay of dietary constitution and cardio-metabolic profile of each subtype [23]. In that sense, the comparison of rapidly available carbohydrates such as grains/grain products, pastries and sweets, displays lower consumption in individuals with diabetes. A similar intake of fresh products like legumes, vegetables and fruits also indicates a health-conscious diet among subjects with diabetes. However, when assessing the intake of animal produce, a rather unfavorable pattern of lower dairy and fish but higher meat consumption can be seen in SIDD- and SIRD-like subtypes. These two subtypes also displayed the highest rate of current smokers, lowest duration of everyday physical activities and highest rates of daytime sleep. Educational status and life satisfaction also differed significantly between subtypes. Again, the SIDD-like and SIRD-like subtypes showed the highest deviation with greater proportions of participants without educational training and lower life satisfaction levels. In return, no differences between subtypes can be seen for physical activity and only minor variation in the gut microbiota composition.

Previous studies on secondary disease risk associated with diabetes subtypes have primarily described complications of nephrotic, hepatic, and retinopathic origin [5, 6]. This data was not available for the FoCus cohort and our analysis therefore focused on common cardio-metabolic comorbidities. This revealed a delayed onset of arterial hypertension and elevated blood lipids in the MARD-like compared to the more severe SIDD-like and SIRD-like subtypes. In contrast to the ANDIS cohort [5, 6], there is no significant difference in subtype-specific cardiovascular risk profiles in our FoCus probands. This is most likely due to pre-existing diabetes with endothelial damage occurring over time regardless of the endotype. Hence, this finding argues for an early and targeted intervention.

Altogether, the MOD-like subtype shows only minor deviations in the lifestyle compared to controls, suggesting moderate interventions to enhance effective weight reduction and metabolic control [24], e.g. by focusing on long-term adherence through a moderate calorie deficit while maintaining a healthful dietary pattern and favorable gut microbiota, and increasing physical activity [25]. In return, our observations propose a stronger need for lifestyle intervention and behavioral adaptation in the severe SIDD- and SIRD-like subtypes. While also aiming at a calorie deficit, the reduction of meat intake, increase of physical activity, smoking cessation and improvement of sleep quality could be specific targets of subtype-adapted training, supporting not only weight loss but also the improvement of insulin sensitivity, microbial composition and overall cardio-metabolic health [26, 27]. Moreover, lower socio-economic status has previously been linked to disease susceptibility and -severity [28], which lines up with our observations. Education and life satisfaction can also impact self-care behaviors, particularly the lifestyle, and access to healthcare resources [29, 30] suggesting that the consideration of these aspects in disease management, e.g. by closer monitoring of treatment adherence, could be beneficial for the affected SIDD- and SIRD-like subtypes. Finally, the MARD-like subtype displayed the lowest BMI among subtypes, with only minor lifestyle variation in physical activity and alcohol intake. These could be specific first-line treatment targets; however it should be considered that older individuals might be limited in the implementation due to impaired mobility or other living conditions leading to quicker exhaustion of lifestyle therapeutic potential.

Despite these promising findings, our study has some limitations. The criteria used to classify subtypes varied slightly from those in the ANDIS cohort due to differences in data availability and cohort characteristics. This variation might have influenced the subtype categorization and comparability, e.g. missing antibody titer information could have influenced the differentiation between SAID and SIDD subtypes. The focus on pre-existing diabetes and obesity, as well as the small sample size for some subtypes (e.g., SAID-like) may reduce generalizability to newly diagnosed or diverse populations. Additionally, the cross-sectional nature of the FoCus cohort limits the ability to assume causal relationships as well as the evaluation of subtype stability over time. Future longitudinal studies are needed to validate these subtypes and explore their development.

## Conclusions

The adaptation of the ANDIS classification framework to the FoCus cohort reveals significant subtype-specific differences in metabolic markers, gut microbiota, diet, physical activity, and social factors. By considering these important facets, our study not only highlights the heterogeneity of diabetes but also supports the development of personalized, subtype-specific therapeutic approaches. Future research should validate these subtypes in larger, diverse populations and explore targeted interventions to optimize diabetes care.

## Supplementary Information

Supplementary Material 1

## Data Availability

All data are available upon request from the P2N biobank. Access token: P2N_I46QR; http://www.uksh.de/p2n/. P2N is a controlled-access human data repository subject to European data protection laws. Therefore, data access is subject to an application, ethics approval by the applicant’s ethics board and a data access agreement.

## Abbreviations

ASV: Amplicon sequencing variant
CRP: C-reactive protein
FoCus: Food chain plus
GLP-1: Glucagon-like peptide 1
MARD: Moderate age-related diabetes
MOD: Moderate obesity-related diabetes
SAID: Severe autoimmune-related diabetes
sDDP-4: Soluble Dipeptidylpeptidase-4
SIDD: Severe insulin-deficient diabetes
SIRD: Severe insulin resistant diabetes
SFRP5: Secreted frizzled related protein 5
UKSH: University Medical Center Schleswig–Holstein
WNT5a: WNT family member 5A
