# Supplementary material for: Comprehensive evaluation of diabetes subtypes in a European cohort reveals stronger differences of lifestyle, education and psychosocial parameters compared to metabolic or inflammatory factors
Source: Cardiovasc Diabetol. 2025 Feb 28;24:99. doi: 10.1186/s12933-025-02660-5 (PMC11871841; doi:10.1186/s12933-025-02660-5)
Supplement: Supplementary file 1 — Supplementary Material 1 [file 12933_2025_2660_MOESM1_ESM.pdf]

Supplement

Characterization of the identified diabetes subtypes in comparison to their respective control groups.

Table S1: Characterization of the SAID-like subtype in comparison to their respective control group.

	SAID-like subtype	control subjects	p-value[a]
**Subjects, n (%)**	6	6	-
**Female sex, n (%)**	3 (50)	4 (75)	0.61
**Age at examination, years**	48.00 [45.25; 51.75]	50.00 [46.00; 61.00]	0.49
**BMI, kg/m^2**	33.88 [25.03; 45.81]	22.58 [21.75; 23.64]	4.66×10^{-3}
**Waist measure, cm**	90 [84.75; 101.25]	78 [74.75; 81.25]	2.82×10^{-2}
**Waist-hip-ratio**	0.84 [0.8; 0.89]	0.78 [0.73; 0.84]	0.14
**Glucose, mg/dl**	189 [174; 282]	91 [88; 96.61]	4.66×10^{-3}
**Insulin, µU/l**	0.3 [0.2; 0.7]	5.9 [4.47; 7.35]	4.25×10^{-2}
**HOMA-IR**	0.21 [0.09; 0.54]	1.35 [1.05; 1.6]	4.26×10^{-2}
**HOMA-beta**	0.81 [0.59; 1.1]	66.78 [53.05; 91.61]	4.66×10^{-3}
**Triglycerides, mmol/l**	116.5 [74.25; 149.25]	73 [63.75; 96.75]	0.28
**Total cholesterol, mmol/l**	4.66 [4.4; 5.19]	-	-
**CRP, mg/l**	1.8 [0.8; 5.19]	-	-
**IL-6, pg/ml**	4.1 [1.98; 5.33]	2.9 [2.03; 3.37]	0.34
**WNT5a, pg/ml**	0.48 [0.2; 0.56]	-	-
**SFRP5, pg/ml**	3.85 [3.44 - 5.25]	-	-
**GLP-1, pg/ml**	4.83 [3.48; 7.22]	3.62 [2.77; 4.53]	0.24

Continuous data is displayed as median [Interquartile Range]; female sex as number (percentage). [a]Statistical significance between the SAID subtype and the control group was tested using Chi2-tests for categorical variables and Kruskal-Wallis-tests for continuous variables. Abbreviations: BMI, Body Mass Index; HOMA-IR, Homeostatic Model Assessment of Insulin Resistance; HOMA-beta, Homeostatic Model Assessment of beta-cell function; CRP, C-reactive protein; IL-6, interleukin 6; Wnt5a, WNT Family Member 5a; SFRP5, secreted frizzled related protein 5; GLP-1, Glucagon-like peptide 1.

Table S2: Characterization of the SIDD-like subtype in comparison to their respective control group.

	SIDD-like subtype	control subjects	p-value[a]
**Subjects, n (%)**	64	64	-
**Female sex, n (%)**	32 (50)	45 (71.88)	3.03×10^{-2}
**Age at examination, years**	53.5 [47; 59.25]	51 [46; 63.25]	0.7
**BMI, kg/m^2**	42.17 [33.04; 50.38]	22.56 [21.63; 23.47]	$< 10^{-5}$
**Waist measure, cm**	118 [110; 131]	80 [76; 86]	$< 10^{-5}$
**Waist-hip-ratio**	0.96 [0.92; 1.01]	0.81 [0.77; 0.85]	$< 10^{-5}$
**Glucose, mg/dl**	151.5 [124.; 207.5]	91 [87; 95]	$< 10^{-5}$
**Insulin, µU/l**	29.25 [14.73; 53.33]	5.00 [3.9; 6.6]	$< 10^{-5}$
**HOMA-IR**	12.26 [5.92; 24.35]	1.19 [0.84; 1.48]	$< 10^{-5}$
**HOMA-beta**	95.05 [64.56; 192.16]	70.16 [54; 88.3]	$< 10^{-5}$
**Triglycerides, mg/dl**	197.5 [120.25; 275.5]	70.5 [54.75; 87]	$< 10^{-5}$
**Total cholesterol, mmol/l**	4.41 [3.74; 5.04]	4.42 [4; 4.58]	0.25
**CRP, mg/l**	5.65 [2.65; 11.32]	0.8 [0.8; 0.8]	$< 10^{-5}$
**IL-6, pg/ml**	5.05 [3.27; 8.2]	1.9 [1.5; 2.95]	$< 10^{-5}$
**WNT5a, pg/ml**	0.39 [0.29; 0.59]	0.32 [0.24; 0.5]	0.19
**SFRP5, pg/ml**	8.26 [1.31; 20.98]	7.03 [2.21; 19.85]	0.84
**GLP-1, pg/ml**	11.75 [4.18; 15.86]	4.33 [2.66; 6.17]	$< 10^{-5}$
**sDPP-4, ng/ml**	543.55 [405.02; 728.47]	512.6 [450.51; 652.56]	0.8

Continuous data is displayed as median [Interquartile Range]; female sex as number (percentage). [a]Statistical significance between the SIDD subtype and the control group was tested using Chi2-tests for categorical variables and Kruskal-Wallis-tests for continuous variables. Abbreviations: BMI, Body Mass Index; HOMA-IR, Homeostatic Model Assessment of Insulin Resistance; HOMA-beta, Homeostatic Model Assessment of beta-cell function; CRP, C-reactive protein; IL-6, interleukin 6; Wnt5a, WNT Family Member 5a; SFRP5, secreted frizzled related protein 5; GLP-1, Glucagon-like peptide 1; sDPP-4, soluble Dipeptidylpeptidase 4.

Table S3: Characterization of the SIRD-like subtype in comparison to their respective control group.

	SIRD-like subtype	control subjects	p-value[a]
**Subjects, n (%)**	66	66	-
**Female sex, n (%)**	36 (54.38)	47 (73.85)	0.07
**Age at examination, years**	55 [44; 64]	53 [48; 65]	0.94
**BMI, kg/m^2**	41.85 [32.75; 50.25]	22.26 [20.79; 23.63]	$< 10^{-5}$
**Waist measure, cm**	122 [110; 137]	81 [75; 88]	$< 10^{-5}$
**Waist-hip-ratio**	0.98 [0.93; 1.02]	0.79 [0.76; 0.87]	$< 10^{-5}$
**Glucose, mg/dl**	121 [109; 162]	91 [86; 95]	$< 10^{-5}$
**Insulin, µU/l**	34.9 [20.8; 50.9]	5.7 [4.5; 7]	$< 10^{-5}$
**HOMA-IR**	10.92 [5.85; 16.87]	1.27 [1.03; 1.64]	$< 10^{-5}$
**HOMA-beta**	174.46 [101.45; 341.18]	72 [57; 88.71]	$< 10^{-5}$
**Triglycerides, mg/dl**	171 [135; 214]	84 [67; 101]	$< 10^{-5}$
**Total cholesterol, mmol/l**	4.2 [3.65; 4.65]	4.5 [3.98; 4.75]	0.21
**CRP, mg/l**	4.1 [2; 10.3]	0.8 [0.8; 1.4]	$< 10^{-5}$
**IL-6, pg/ml**	4.9 [3.6; 6.5]	2.4 [1.5; 3.3]	$< 10^{-5}$
**WNT5a, pg/ml**	0.35 [0.24; 0.46]	0.35 [0.27; 0.51]	0.76
**SFRP5, pg/ml**	5.07 [1.32; 28.5]	4.09 [1.81; 11.78]	0.44
**GLP-1, pg/ml**	9.89 [5.14; 15.5]	5.25 [3.65; 7.66]	6.71×10^{-5}
**sDPP-4, ng/ml**	527.23 [402.93; 646.39]	452.05 [350.89; 572.14]	0.14

Continuous data is displayed as median [Interquartile Range]; female sex as number (percentage). [a]Statistical significance between the SIRD subtype and the control group was tested using Chi2-tests for categorical variables and Kruskal-Wallis-tests for continuous variables. Abbreviations: BMI, Body Mass Index; HOMA-IR, Homeostatic Model Assessment of Insulin Resistance; HOMA-beta, Homeostatic Model Assessment of beta-cell function; CRP, C-reactive protein; IL-6, interleukin 6; Wnt5a, WNT Family Member 5a; SFRP5, secreted frizzled related protein 5; GLP-1, Glucagon-like peptide 1; sDPP-4, soluble Dipeptidylpeptidase 4.

Table S4: Characterization of the MOD-like subtype in comparison to their respective control group.

	MOD-like subtype	control subjects	p-value[a]
**Subjects, n (%)**	37	37	-
**Female sex, n (%)**	26 (72.22)	25 (69.44)	1
**Age at examination, years**	60.5 [49.75; 64]	54.5 [47.75; 61.5]	0.08
**BMI, kg/m^2**	38.96 [32.63; 42.88]	22.29 [20.9; 22.95]	$< 10^{-5}$
**Waist measure, cm**	118 [110; 123]	82 [73.88; 86]	$< 10^{-5}$
**Waist-hip-ratio**	0.95 [0.91; 1.01]	0.82 [0.78; 0.86]	$< 10^{-5}$
**Glucose, mg/dl**	102 [93.75; 115.75]	90 [87; 94]	2.48×10^{-5}
**Insulin, µU/l**	14.75 [9.43; 17.28]	5.25 [4; 6.9]	$< 10^{-5}$
**HOMA-IR**	3.71 [2.50; 4.36]	1.16 [0.91; 1.61]	$< 10^{-5}$
**HOMA-beta**	125.22 [75.53; 202.66]	64.99 [53.56; 85.7]	2.1×10^{-5}
**Triglycerides, mg/dl**	122 [102; 147]	74 [58.25; 90.75]	$< 10^{-5}$
**Total cholesterol, mmol/l**	4.27 [3.60; 4.97]	4.61 [4.34; 4.65]	0.55
**CRP, mg/l**	3.5 [1.25; 8]	0.8 [0.8; 0.92]	$< 10^{-5}$
**IL-6, pg/ml**	4.7 [3.2; 7]	2 [1.5; 3.1]	$< 10^{-5}$
**WNT5a, pg/ml**	0.33 [0.22; 0.45]	0.39 [0.34; 0.62]	0.28
**SFRP5, pg/ml**	9.3 [6.19; 15.9]	6.12 [5.79; 12.34]	0.82
**GLP-1, pg/ml**	5.91 [4.16; 12.55]	4.8 [2.79; 8.16]	0.09
**sDPP-4, ng/ml**	527.28 [352.06; 594.10]	448.17 [380.09; 574.32]	0.9

Continuous data is displayed as median [Interquartile Range]; female sex as number (percentage). [a]Statistical significance between the MOD subtype and the control group was tested using Chi2-tests for categorical variables and Kruskal-Wallis-tests for continuous variables. Abbreviations: BMI, Body Mass Index; HOMA-IR, Homeostatic Model Assessment of Insulin Resistance; HOMA-beta, Homeostatic Model Assessment of beta-cell function; CRP, C-reactive protein; IL-6, interleukin 6; Wnt5a, WNT Family Member 5a; SFRP5, secreted frizzled related protein 5; GLP-1, Glucagon-like peptide 1; sDPP-4, soluble Dipeptidylpeptidase 4.

Table S5: Characterization of the MARD-like subtype in comparison to their respective control group.

	MARD-like subtype	control subjects	p-value[a]
**Subjects, n (%)**	35	35	-
**Female sex, n (%)**	20 (57.14)	16 (45.71)	0.47
**Age at examination, years**	70 [65.5; 73]	67 [58; 71]	1.21×10^{-2}
**BMI, kg/m^2**	30.04 [26.32; 36.64]	23.18 [22.3; 23.88]	$< 10^{-5}$
**Waist measure, cm**	107.5 [96; 121.5]	85 [80.5; 92.5]	$< 10^{-5}$
**Waist-hip-ratio**	0.95 [0.9; 1]	0.84 [0.81; 0.9]	$< 10^{-5}$
**Glucose, mg/dl**	118 [106; 138]	94.5 [90.25; 101]	$< 10^{-5}$
**Insulin, µU/l**	17.7 [9.9; 27.25]	6.05 [4.5; 7.2]	$< 10^{-5}$
**HOMA-IR**	5.2 [3.52; 7.92]	1.44 [1.12; 1.74]	$< 10^{-5}$
**HOMA-beta**	111.39 [54.75; 188.22]	68.18 [55.53; 77.94]	5.16×10^{-3}
**Triglycerides, mg/dl**	151 [121.5; 242.5]	75 [64; 90]	$< 10^{-5}$
**Total cholesterol, mmol/l**	4.53 [4.09; 4.93]	4.52 [4; 4.73]	0.71
**CRP, mg/l**	2.3 [0.8; 5.05]	0.9 [0.8; 1.65]	5.52×10^{-3}
**IL-6, pg/ml**	5.2 [3.05; 7]	2.6 [1.55; 3.65]	1.46×10^{-5}
**WNT5a, pg/ml**	0.52 [0.32; 0.58]	0.33 [0.18; 0.49]	0.18
**SFRP5, pg/ml**	8.34 [4.33; 13.06]	7.57 [6.32; 13.55]	1
**GLP-1, pg/ml**	10.31 [3.96; 18.52]	5.25 [3.18; 5.61]	5.78×10^{-3}
**sDPP-4, ng/ml**	575.52 [495.84; 660]	437.13 [383.13; 526.21]	4.56×10^{-2}

Continuous data is displayed as median [Interquartile Range]; female sex as number (percentage). [a]Statistical significance between the MARD subtype and the control group was tested using Chi2-tests for categorical variables and Kruskal-Wallis-tests for continuous variables. Abbreviations: BMI, Body Mass Index; HOMA-IR, Homeostatic Model Assessment of Insulin Resistance; HOMA-beta, Homeostatic Model Assessment of beta-cell function; CRP, C-reactive protein; IL-6, interleukin 6; Wnt5a, WNT Family Member 5a; SFRP5, secreted frizzled related protein 5; GLP-1, Glucagon-like peptide 1; sDPP-4, soluble Dipeptidylpeptidase 4.
